# Trait differentiation and modular toxin expression in palm-pitvipers

**DOI:** 10.1186/s12864-020-6545-9

**Published:** 2020-02-11

**Authors:** Andrew J. Mason, Mark J. Margres, Jason L. Strickland, Darin R. Rokyta, Mahmood Sasa, Christopher L. Parkinson

**Affiliations:** 1 Department of Biological Sciences, Clemson University, 190 Collings St., Clemson, SC, 29634 USA; 2 Department of Biological Sciences, Florida State University, Tallahassee, FL, 24105 USA; 3 Instituto Clodomiro Picado, Facultad de Microbiologia, Universidad de Costa Rica, San Jose, Costa Rica; 4 Department of Forestry, and Environmental Conservation, Clemson University, Clemson, SC, USA

**Keywords:** Modularity, Venom, Gene family evolution, Transcriptomics, *Bothriechis*

## Abstract

**Background:**

Modularity is the tendency for systems to organize into semi-independent units and can be a key to the evolution and diversification of complex biological systems. Snake venoms are highly variable modular systems that exhibit extreme diversification even across very short time scales. One well-studied venom phenotype dichotomy is a trade-off between neurotoxicity versus hemotoxicity that occurs through the high expression of a heterodimeric neurotoxic phospholipase A_2_ (PLA_2_) or snake venom metalloproteinases (SVMPs). We tested whether the variation in these venom phenotypes could occur via variation in regulatory sub-modules through comparative venom gland transcriptomics of representative Black-Speckled Palm-Pitvipers (*Bothriechis nigroviridis*) and Talamancan Palm-Pitvipers (*B. nubestris*).

**Results:**

We assembled 1517 coding sequences, including 43 toxins for *B. nigroviridis* and 1787 coding sequences including 42 toxins for *B. nubestris*. The venom gland transcriptomes were extremely divergent between these two species with one *B. nigroviridis* exhibiting a primarily neurotoxic pattern of expression, both *B. nubestris* expressing primarily hemorrhagic toxins, and a second *B. nigroviridis* exhibiting a mixed expression phenotype. Weighted gene coexpression analyses identified six submodules of transcript expression variation, one of which was highly associated with SVMPs and a second which contained both subunits of the neurotoxic PLA_2_ complex. The sub-module association of these toxins suggest common regulatory pathways underlie the variation in their expression and is consistent with known patterns of inheritance of similar haplotypes in other species. We also find evidence that module associated toxin families show fewer gene duplications and transcript losses between species, but module association did not appear to affect sequence diversification.

**Conclusion:**

Sub-modular regulation of expression likely contributes to the diversification of venom phenotypes within and among species and underscores the role of modularity in facilitating rapid evolution of complex traits.

## Background

Modularity, the tendency for systems to organize into semi-independent discrete units, is a central theme in the evolution of biological systems and complex traits [1]. Modularity creates evolvability and the potential to adapt to novel environments rapidly by eliminating or reducing antagonistic pleiotropy while simultaneously permitting advantageous phenotypic changes through the use of conserved genetic machinery [2, 3]. Gene regulatory networks are an especially common mechanism for modular evolution within and among lineages [4]. Inducing, increasing, reducing, or eliminating expression of specific sub-modules can create or replicate advantageous phenotypes through the recombination of sub-modular features [5]. As such, modularity is a common characteristic of many adaptive traits because sub-module associated features can be rapidly modified without evolving ‘from scratch’ [2]. *Heliconius* butterflies provide a classic example where a variety of predator-deterring wing patterns have evolved and diversified through variation in modular elements (e.g., color and spot-pattern) controlled by just a few conserved genes (e.g., the *optix* transcription factor and the *wntA* signaling pathway) [5–7]. Identifying modules and their sub-modules underlying variation in highly variable modular traits can therefore provide valuable insight on the genetic basis of diversification across micro and macroscales.

Snake venoms are highly variable adaptive traits composed of 10–100 secreted proteins that collectively work to subdue prey or deter predation [8, 9]. Despite the perceived complexity of the venom system, venoms appear to evolve rapidly and respond to local selection pressures over short timescales [10, 11]. The exceptional degree of phenotypic variation observed in venoms can partially be contributed to the modularity of the venom system. Because toxin expression and production is localized to a specialized gland [12–15] (but see [16, 17]), the venom system is a functional module that is inherently more free to vary with limited pleiotropic effects. Moreover, venom functionality is, at least in part, dependent on the coordinated expression of specific toxins or toxin classes which may covary geographically or among species [18–20]. In many cases, recurrent patterns of variation in venom compositions suggest that expression of associated toxins represent sub-modules of variation, though empirical tests of sub-modularity of toxins are lacking.

One example of venom variation likely mediated by sub-modular regulation is an apparent phenotypic trade-off between neurotoxicity and hemotoxicity. In crotalid vipers (*Viperidae: Crotalinae*), hemorrhagic venoms are most common and are a function of high proportions of several toxin families, especially snake venom metalloproteinases (SVMPs) [21, 22]. However, in some lineages neurotoxicity has emerged as a principal phenotype [22]. An extremely well-documented manifestation of neurotoxicity in crotalid venoms is based on high expression of a heterodimeric *β*-neurotoxic phospholipase A_2_ (PLA_2_) complex [23, 24]. These phenotypes can manifest as interspecific, intraspecific, and/or ontogenetic variation [18–20, 22, 25–28], prompting the establishment of a “Type A/Type B" nomenclature to describe the variation in rattlesnakes. Type A venoms refer to those dominated by the neurotoxic PLA_2_s, and Type B venoms refer to those with high proportions of SVMPs. Notably, there are also descriptions of Type A+B venoms which have high proportions of neurotoxic PLA_2_s and hemorrhagic SVMPs, but these phenotypes are rare even in Type A - Type B contact zones [11, 19, 29]. Here, recurring phenotypic patterns, the lack of apparent phylogenetic signal (even over ecological time scales), and the usage of common genetic building blocks (i.e., toxin families) is suggestive of modularity mediating the evolution of these phenotypes.

An opportunity to test this exists in the arboreal pitvipers of the genus *Bothriechis*. One species, *B. nigroviridis*, exhibits a neurotoxic venom phenotype driven by the high abundance of a neurotoxic heterodimeric PLA_2_ named nigroviriditoxin [30, 31]. *Bothriechis nigroviridis* is unique among species with neurotoxic venom because of its ecological differentiation; *B. nigroviridis* is an arboreal high-elevation specialist while most others are mid-low elevation terrestrial species. The sister species to *B. nigroviridis*, *B. nubestris*, appears to occupy an extremely similar ecological niche based on its documented range and conserved morphology [32]. Although empirical studies of *B.nubestris’* venom have yet to be conducted, its divergence from *B. nigroviridis* 6–10 mya would provide sufficient temporal opportunity for venom diversification [33]. *Bothriechis nigroviridis* and *B. nubestris* can therefore provide a test case for examining mechanisms of phenotypic diversification in a modular framework.

We sought to describe and compare the venom gland transcriptomes of *B. nigroviridis* and *B. nubestris* to understand toxin evolution in a modular framework. We characterize the venom gland transcriptomes of representatives of each species and identify key dimensions of variation within and between species. We identified conserved and unique toxins and used weighted-gene co-expression network analysis (WGCNA) to test for sub-modules of variation among distinct venom types. Based on the observation that neurotoxic and hemotoxic phenotypes occur independently, in combination, or as ontogenetic changes, we hypothesized that toxins associated with neurotoxic and hemorrhagic phenotypes (i.e., neurotoxic PLA_2_s and SVMPs) would segregate into distinct sub-modules of correlated expression variation. Additionally, we examine instances of intraspecific transcript duplication and loss and comparative sequence divergence. We hypothesized that if modular expression is a primary driver of variation, gene duplications and sequence diversification would be reduced in sub-module associated toxin families whose function has been selectively optimized and is primarily regulated by expression.

## Results

### Transcriptome characterization

**Fig. 1:**
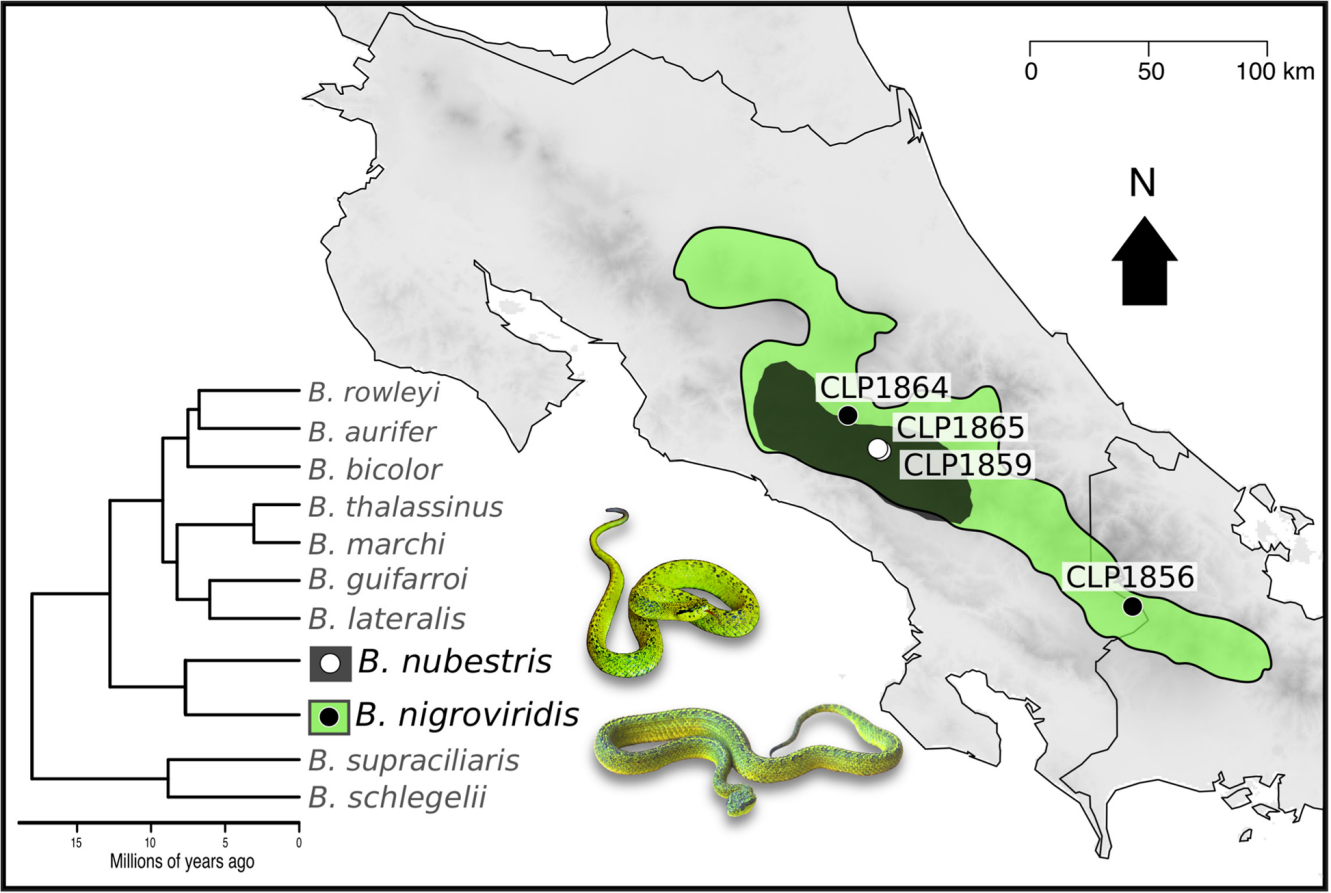
Phylogeny of *Bothriechis* based on [33] and a distribution map for *B. nigroviridis* and *B. nubestris* made in R v.3.5.3 (https://www.R-project.org/) based on ranges described in [74] and [33] and publicly available specimen localities in [32]. Sampled localities are shown as dots with specimen labels. Animal images were modified and used with permission from credit holder Alexander Robertson

To examine the evolutionary mechanisms underlying venom divergence we sequenced, assembled, and characterized the venom gland transcriptomes of two *Bothriechis nigroviridis* (CLP1856 and CLP1864) and two *B. nubestris* (CLP1859 and CLP1865) (Fig. 1, Table 1). The number of recovered toxins and recovered families were generally consistent with those of other viperid transcriptomes [25, 34–37] and with estimates of toxin family size in early high-throughput transcriptomes of *B. schlegelii* and *B. lateralis* [38] (Table 2, Table 3).

**Table 1:** Specimen information for *Bothriechis* individuals used in this work

Species	Specimen ID	Museum ID	Total Reads	Merged	Total Unique CDS	CDS Passing QC	SRR
*B. nigroviridis*	CLP1856	MZUCR23264	20002019	17227317	3177	807	SRR9968896
*B. nigroviridis*	CLP1864	MZUCR23270	24641535	21035386	3323	1416	SRR9968897
*B. nubestris*	CLP1859	MZUCR23267	20628335	16855601	3125	1476	SRR9968894
*B. nubestris*	CLP1865	MZUCR23271	23443610	20108934	3297	1461	SRR9968895

**Table 2:** Toxin transcripts recovered for *Bothriechis nigroviridis* and associated classifications as orthologs or paralogs, expected transcripts per million reads (TPM) estimated by RSEM, likely over expression classification as detected in intraspecific variation comparisons (i.e., above the 99th percentile of expected variance in expression based on a nontoxin null distribution), and coverage-based assessment of likely presence or absence

Toxin ID	Ortholog/ Paraloge	TPM		Likely Over Expression	Presence/Absence	
		CLP1856	CLP1864		CLP1856	CLP1864
Bnigro-BPP-1	Ortholog	29752.98	52897.54	CLP1864	+	+
Bnigro-CTL-1	Ortholog	5601.97	3.99	CLP1856	+	-
Bnigro-CTL-2	Ortholog	47.42	4353.63	CLP1864	-	+
Bnigro-CTL-3	Ortholog	4669.5	3027.69	-	+	+
Bnigro-CTL-4	Paralog	5395.92	4095.4	-	+	+
Bnigro-CTL-5	Paralog	7653.39	26592.73	CLP1864	+	+
Bnigro-CTL-6	Paralog	8809.55	3537.01	-	+	+
Bnigro-CTL-7	Paralog	0	22739.95	CLP1864	-	+
Bnigro-HYAL-1	Ortholog	196.31	76.03	-	+	+
Bnigro-LAAO-1	Ortholog	2070.21	412.19	CLP1856	+	+
Bnigro-NGF-1	Ortholog	836.47	1692.1	-	+	+
Bnigro-NUC-1	Ortholog	1575.76	1532.59	-	+	+
Bnigro-PDE-1	Ortholog	1356.81	524.9	-	+	+
Bnigro-PLA_2_-1	Ortholog	53023.62	183732.85	CLP1864	+	+
Bnigro-PLA_2_-2	Ortholog	102035.05	235935.29	CLP1864	+	+
Bnigro-SVMPII-1	Ortholog	3405.08	327.07	CLP1856	+	-
Bnigro-SVMPII-2	Ortholog	4055.07	290.21	CLP1856	+	-
Bnigro-SVMPII-3	Ortholog	3980.17	24.59	CLP1856	+	-
Bnigro-SVMPII-4	Paralog	52404.14	11942.88	-	+	+
Bnigro-SVMPIII-1	Ortholog	0.68	73.09	CLP1864	-	+
Bnigro-SVMPIII-2	Ortholog	2157.48	151.17	CLP1856	+	+
Bnigro-SVMPIII-3	Ortholog	12908.06	124.4	CLP1856	+	+
Bnigro-SVMPIII-4	Ortholog	6587.36	2375.96	CLP1856	+	-
Bnigro-SVMPIII-5	Ortholog	48324.54	19456.86	-	+	+
Bnigro-SVSP-1	Ortholog	5067.97	24092.29	CLP1864	+	+
Bnigro-SVSP-2	Ortholog	4588.27	3441.02	-	+	+
Bnigro-SVSP-3	Ortholog	1633.58	4877.37	CLP1864	+	+
Bnigro-SVSP-4	Ortholog	1174.85	19016.69	CLP1864	+	+
Bnigro-SVSP-5	Ortholog	552.15	644.59	-	+	+
Bnigro-SVSP-6	Ortholog	1712.44	8567.09	CLP1864	-	+
Bnigro-SVSP-7	Ortholog	792.67	4112.06	CLP1864	+	+
Bnigro-SVSP-8	Ortholog	17931.94	0.41	CLP1856	+	-
Bnigro-SVSP-9	Paralog	11.35	25032.15	CLP1864	-	+
Bnigro-SVSP-10	Paralog	183.43	2875.45	CLP1864	+	+
Bnigro-SVSP-11	Paralog	2827.4	0.16	CLP1856	+	-
Bnigro-SVSP-12	Paralog	3.89	8988.68	CLP1864	-	+
Bnigro-SVSP-13	Paralog	21317.45	0	CLP1856	+	-
Bnigro-VEGF-1	Ortholog	56.56	17963.5	CLP1864	+	+
Bnigro-VEGF-2	Ortholog	33.93	117.99	-	+	+
Bnigro-VEGF-3	Ortholog	14.81	62.5	-	+	+
Bnigro-VEGF-4	Ortholog	25.22	77.81	-	+	+
Bnigro-Vespryn-1	Paralog	8.98	45.01	-	+	+
Bnigro-Waprin-1	Ortholog	24.92	36.31	-	+	+

**Table 3:** Toxin transcripts recovered for *Bothriechis nubestris* and associated classifications as orthologs or paralogs, expected transcripts per million reads (TPM) estimated by RSEM, over expression classification as detected in intraspecific variation comparisons (i.e., above the 99th percentile of expected variance in expression based on a nontoxin null distribution), and coverage-based assessment of likely presence or absence

Toxin ID	Ortholog/ Paralog	TPM		Likely Over Expression	Presence/Absence	
		CLP1859	CLP1865		CLP1859	CLP1865
Bnubes-BPP-1	Ortholog	5097.77	63484.01	CLP1865	-	+
Bnubes-CRISP-1	Paralog	17682.06	8634.01	CLP1859	+	+
Bnubes-CTL-1	Ortholog	48790.4	45771.89	-	+	+
Bnubes-CTL-2	Ortholog	13469.89	9134.83	-	+	+
Bnubes-CTL-3	Ortholog	18273.91	8462.73	CLP1859	+	+
Bnubes-CTL-4	Paralog	134247.25	46096.08	CLP1859	+	+
Bnubes-CTL-5	Paralog	93992.03	44194.92	CLP1859	+	+
Bnubes-CTL-6	Paralog	41975.1	23098.03	CLP1859	+	+
Bnubes-HYAL-1	Ortholog	312.56	480.7	-	+	+
Bnubes-KUN-1	Paralog	211.54	231.29	-	+	+
Bnubes-LAAO-1	Ortholog	6946.11	12529.4	-	+	+
Bnubes-NGF-1	Ortholog	3347.21	5435.31	-	+	+
Bnubes-NUC-1	Ortholog	1184.42	2318.22	-	+	+
Bnubes-PDE-1	Ortholog	1156.15	1461.55	-	+	+
Bnubes-PLA_2_-1	Ortholog	3073.31	3700.01	-	+	+
Bnubes-PLA_2_-2	Ortholog	2321.73	209.08	CLP1859	+	+
Bnubes-PLA_2_-3	Paralog	4646.1	91726.32	CLP1865	+	+
Bnubes-SVMPII-1	Ortholog	11115.83	7867.18	-	+	+
Bnubes-SVMPII-2	Ortholog	7446.39	7182.3	-	+	+
Bnubes-SVMPII-3	Ortholog	85.26	6966.13	CLP1865	+	+
Bnubes-SVMPII-4	Paralog	9408.28	9519.11	-	+	+
Bnubes-SVMPII-5	Paralog	72976.3	52932.86	-	+	+
Bnubes-SVMPIII-1	Ortholog	4.02	52.08	CLP1865	-	+
Bnubes-SVMPIII-2	Ortholog	7436.41	6075.36	-	+	+
Bnubes-SVMPIII-3	Ortholog	14334.66	14644.25	-	+	+
Bnubes-SVMPIII-4	Ortholog	6744.23	10192.23	-	+	+
Bnubes-SVMPIII-5	Ortholog	131295.22	69281.92	CLP1859	+	+
Bnubes-SVMPIII-6	Paralog	808.43	2990.11	-	+	+
Bnubes-SVSP-1	Ortholog	5793.23	2477.48	CLP1859	+	+
Bnubes-SVSP-2	Ortholog	1544.31	2924.28	-	+	+
Bnubes-SVSP-3	Ortholog	3126.56	3125.05	-	+	+
Bnubes-SVSP-4	Ortholog	7665.15	2252.2	CLP1859	+	+
Bnubes-SVSP-5	Ortholog	2301.11	4094.43	-	+	+
Bnubes-SVSP-6	Ortholog	5123.44	2684.33	-	+	+
Bnubes-SVSP-7	Ortholog	795.14	393.28	-	+	+
Bnubes-SVSP-8	Ortholog	3207.97	10487.13	-	+	+
Bnubes-SVSP-9	Paralog	823.13	475.48	-	+	+
Bnubes-VEGF-1	Ortholog	3542.02	413.99	CLP1859	+	+
Bnubes-VEGF-2	Ortholog	222.72	119.16	-	+	+
Bnubes-VEGF-3	Ortholog	109.03	51.22	-	+	+
Bnubes-VEGF-4	Ortholog	61.69	68.27	-	+	+
Bnubes-Waprin-1	Ortholog	28.73	25.35	-	+	+

**Fig. 2:**
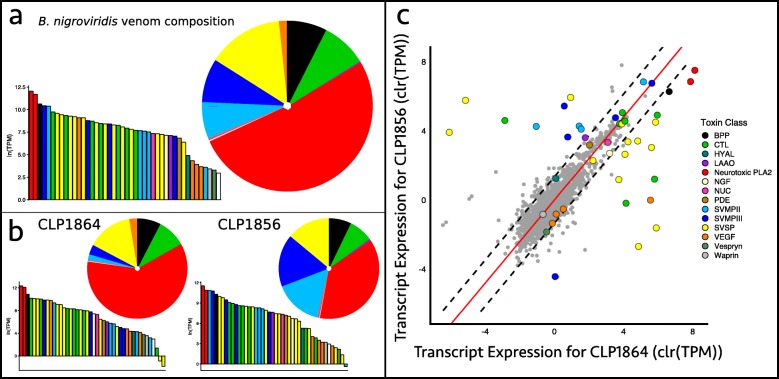
Venom characterization for *Bothriechis nigroviridis*. **a** Venom transcriptome compositions for *B. nigroviridis* based on average expression between two individuals. **b** Venom transcriptome compositions of each individual used. The venom of *B. nigroviridis* CLP1864 is largely consistent with the published proteome for this species. The high proportion of snake venom metalloproteinases (SVMPs) observed in the venom gland transcriptome of *B. nigroviridis* CLP1856 has not been described previously. **c** Intraspecific variation in transcript expression for *B. nigroviridis*. Data have been centered log-ratio transformed to account for their compositional nature. Dashed lines denote the 99% confidence interval of nontoxin expression and red lines are lines of best fit based on orthogonal residuals. *B. nigroviridis* displays substantially more variation in toxin expression, primarily in C-type lectins (CTLs), SVMPs, and snake venom serine proteinases (SVSP)s

We recovered 1517 total transcripts for *B. nigroviridis*, which included 43 toxins from 13 toxin families. The venom transcriptome of *B. nigroviridis* was largely dominated by the expression of the heterodimeric neurotoxic PLA_2_, nigroviriditoxin [31], especially in the northern individual where it accounted for 60.3% of toxin expression (Fig. 2, Table 2). BPPs and SVSPs were also abundant in *B. nigroviridis* venoms, accounting for 7.6% and 14.6% of toxin expression, respectively (Fig. 2, Table 2). The high expression of the neurotoxic PLA_2_ complex observed in the northern individual is consistent with the neurotoxic phenotype previously described in individuals from a similar locality (∼50 km north of CLP1864’s locality, though from a different cordillera) [30] (Type A based on the rattlesnake nomenclature). Consistent with the Type A phenotype, there was low expression of CTL and SVMP variants which, in a previous proteomic study of *B. nigroviridis*, were not detected in the venom [30].

Unlike the northern *B. nigroviridis*, the southern *B. nigroviridis* showed substantial expression of the nigroviriditoxin subunits as well as SVMPs (Fig. 2, Table 2). Both subunits of nigroviriditoxin and seven of the nine SVMPS were identified as outliers in expression comparisons between the two individuals; nigroviriditoxin and one SVMP were found to be expressed outside of the 99th percentile of the null distribution in the northern *B. nigroviridis* while six SVMPs were expressed outside of the 99th percentile of the null distribution in the southern *B. nigroviridis* (Table 2). In addition to the toxin family differences, four CTL and 11 SVSP variants fell outside of the 99th percentile of the null distribution of expression divergence between individuals (Table 2). Of the 43 total toxins assembled for *B. nigroviridis*, 27 were expressed outside of the 99th percentile of the nontoxin null distribution. In many cases, expression differences could be explained by toxin absence. Overall, 14 toxins were found to be absent in one individual with six absences in the southern *B. nigroviridis* and eight absences in the northern *B. nigroviridis*. The overall pattern of toxin expression is more characteristic of a Type A+B phenotype than Type A [39].

**Fig. 3:**
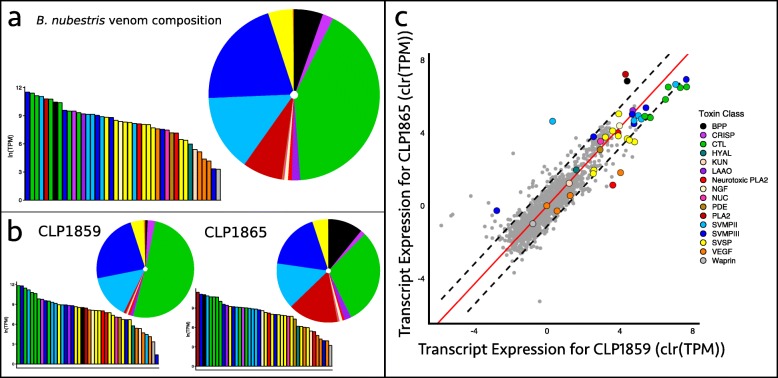
Venom characterization for *Bothriechis nubestris*. **a** Venom transcriptome compositions for *B. nubestris* based on average expression between two individuals for each species. **b** Venom transcriptome compositions of each individual used. The venom of *B. nubestris* is dominated by SVMPs and CTLs. **c** Intraspecific variation in transcript expression for *B. nubestris*. Data have been centered log-ratio transformed to account for their compositional nature. Dashed lines denote the 99% confidence interval of nontoxin expression and red lines are lines of best fit based on orthogonal residuals. The venoms of *B. nubestris* CLP1859 and CLP1865 are largely similar, though CLP1865 displays elevated expression of a basic PLA_2_ and BPPs

For *B. nubestris* we recovered 1787 transcripts which included 42 toxins from 14 toxin families (Table 3). In contrast to *B. nigroviridis*, toxin expression and presences/absences were generally similar between the two sequenced individuals of *B. nubestris* (Fig. 3, Table 3). In total, 14 toxins were expressed outside of the 99th percentile of the nontoxin null distribution. Toxins whose expression was outside the 99th percentile spanned all major families including BPP, CTLs, PLA_2_s, SVMPs, and SVSPs. However, only two toxins, Bnube-BPP-1 and Bnube-SVMPIII-1, were found to be absent in one individual. The overall expression pattern for both individuals was broadly consistent with observed Type B venoms [18]. SVMPs and CTLs were highly abundant components in the venom making up, on average 34.9% and 40.4% of toxin expression, respectively. In addition to SVMPs and CTLs, *B. nubestris* also expressed three PLA_2_s at lower levels. Two of these PLA_2_s were orthologous to the alpha and beta subunits of nigroviriditoxin on average accounting for 0.2% and 0.5% of toxin expression, respectively. The third PLA_2_, Bnube-PLA2-3, made up 15.7% of toxin expression in one *B. nubestris* individual (CLP1865) and appears homologous to a non-enzymatic, myotoxic PLA_2_ in *B. schlegelii* [40, 41].

### Interspecific variation and submodule identification

**Fig. 4:**
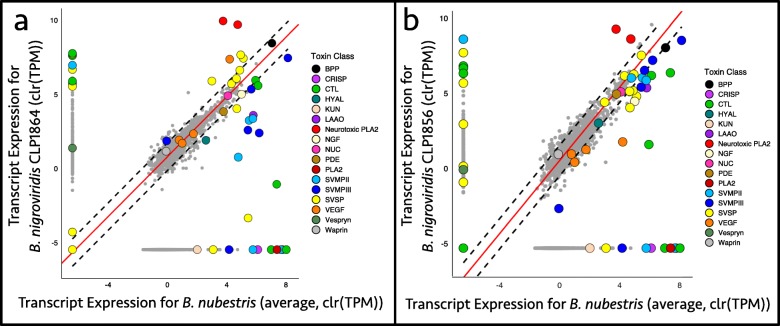
Interspecific comparisons of toxin expression between average *Bothriechis nubestris* toxin expression and **a** Type A *B. nigroviridis* and **b** Type A+B *B. nigroviridis*. TPM values have been centered log-ratio transformed to account for the compositional nature of the data. Dashed lines denote the 99% confidence interval of nontoxin expression and red lines are lines of best fit based on orthogonal residuals. Paralogs are shown near axes for each species

OrthoFinder [42] identified 1282 one-to-one orthologs, which included 32 orthologous toxins. Due to the high variability in toxin expression observed between individuals of *B. nigroviridis*, we compared toxin expression of each individual to the average expression of *B. nubestris* (Fig. 4). High variation in ortholog expression was observed between the northern *B. nigroviridis* and *B. nubestris*, with 14 toxins detected as differentially expressed by DESeq2 (Fig. 4, Table 4). The most prominent pattern was the variation in expression of nigroviriditoxin subunits and SVMPs (Fig. 4); a pattern which supports the classification of the northern *B. nigroviridis’* venom as Type A and *B. nubestris’* venom as Type B. In contrast, only 8 orthologous toxins were detected as differentially expressed between the southern *B. nigroviridis* and *B. nubestris* (Fig. 4, Table 5). Moreover, the variance in orthologous expression between the southern *B.nigroviridis* and *B. nubestris* was substantially lower than observed in the previous comparison, due largely to increased expression of several SVMPs.

**Table 4:** DESeq2 expression analyses for *B. nigroviridis* A versus *B. nubestris* toxins comparison

Toxin	baseMean	log2FoldChange	lfcSE	stat	p-value	p-adj
SVSP-4	56149.550	-2.041	0.790	-2.582	0.010	0.136
SVSP-1	62813.029	-2.650	0.676	-3.920	<0.001**	0.003**
SVSP-2	14843.268	-0.759	0.651	-1.166	0.243	0.819
SVSP-6	31232.805	-1.245	0.643	-1.935	0.053	0.405
SVSP-7	10256.001	-2.901	0.713	-4.069	<0.001**	0.002**
SVSP-3	21197.683	-0.765	0.534	-1.433	0.152	0.685
SVSP-8	24729.723	13.915	1.148	12.121	<0.001**	<0.001**
VEGF-2	575.456	0.408	0.512	0.798	0.425	0.968
VEGF-4	176.204	-0.412	0.432	-0.953	0.341	0.913
VEGF-3	329.392	0.240	0.558	0.429	0.668	0.983
CTL-2	21966.999	1.227	0.588	2.087	0.037	0.322
CTL-1	75597.100	13.412	0.619	21.666	<0.001**	<0.001**
SVMPII-3	26046.537	7.004	3.014	2.324	0.020*	0.228
SVMPII-2	58055.510	4.545	0.471	9.658	<0.001**	<0.001**
CTL-3	27445.563	2.004	0.682	2.940	0.003**	0.061
LAAO-1	81941.767	4.437	0.564	7.862	<0.001**	<0.001**
NGF-1	17559.052	1.241	0.606	2.047	0.041	0.350
NUC-1	24635.841	0.0662	0.630	0.105	0.916	0.999
PDE-1	22815.921	1.204	0.537	2.242	0.025*	0.258
PLA_2_-1	153799.239	-5.920	0.508	-11.652	<0.001**	<0.001***
PLA_2_-2	193031.819	-7.691	1.132	-6.796	<0.001**	<0.001***
SVMPIII-5	1120896.609	2.273	0.848	2.681	0.007	0.115
SVMPIII-3	146258.094	6.755	0.495	13.645	<0.001**	<0.001**
SVMPIII-2	68851.762	5.381	0.499	10.779	<0.001**	<0.001**
SVMPIII-4	96830.991	1.714	0.522	3.286	0.001**	0.025*
SVSP-5	12727.207	2.176	0.638	3.408	0.001**	0.019*
SVMPII-1	75173.762	4.756	0.534	8.899	<0.001**	<0.001**
BPP-1	162148.870	-0.756	1.137	-0.665	0.506	0.982
HYAL-1	3084.833	2.260	0.587	3.853	<0.001**	0.004**
SVMPIII-1	676.400	-1.536	2.179	-0.705	0.481	0.982
VEGF-1	19141.242	-3.323	1.095	-3.036	0.002**	0.050
Waprin-1	65.350	-0.587	0.626	-0.938	0.348	0.915

Statistically significant *p*-values are denoted with asterisks

**Table 5:** DESeq2 expression analyses for *B. nigroviridis* A + B versus *B. nubestris* toxins comparison

Toxin	baseMean	log2FoldChange	lfcSE	stat	*p*-value	*p*-adj
SVSP-4	17900.673	1.529	0.879	1.740	0.082	0.327
SVSP-1	23976.545	-0.846	0.744	-1.136	0.256	0.562
SVSP-2	17011.775	-1.609	0.720	-2.234	0.025*	0.171
SVSP-6	15736.775	0.634	0.743	0.854	0.393	0.704
SVSP-7	3569.069	-0.970	0.718	-1.351	0.177	0.483
SVSP-3	13267.220	0.373	0.656	0.569	0.570	0.805
SVSP-8	61332.390	-1.966	0.681	-2.886	0.004**	0.053
VEGF-2	398.593	1.786	0.519	3.445	0.001**	0.012*
VEGF-4	113.051	0.845	0.535	1.580	0.114	0.391
VEGF-3	218.467	1.899	0.590	3.220	0.001**	0.021*
CTL-2	15138.097	7.366	0.698	10.557	<0.001**	<0.001**
CTL-1	68552.480	2.542	0.432	5.890	<0.001**	<0.001**
SVMPII-3	40750.244	-0.784	3.020	-0.260	0.795	0.908
SVMPII-2	67043.525	0.277	0.433	0.641	0.522	0.780
CTL-3	25464.314	0.979	0.707	1.384	0.166	0.469
LAAO-1	77794.353	1.648	0.514	3.206	0.001**	0.022*
NGF-1	13847.466	1.826	0.701	2.606	0.009**	0.088
NUC-1	23423.714	-0.436	0.683	-0.638	0.523	0.782
PDE-1	27866.068	-0.637	0.571	-1.115	0.265	0.572
PLA_2_-1	50595.962	-4.499	0.465	-9.673	<0.001**	<0.001***
PLA_2_-2	90982.168	-6.867	2.083	-3.296	0.001**	0.018*
SVMPIII-5	1147158.941	0.489	0.697	0.702	0.483	0.766
SVMPIII-3	202400.932	-0.410	0.438	-0.937	0.349	0.661
SVMPIII-2	70220.557	1.077	0.453	2.377	0.017*	0.134
SVMPIII-4	111332.469	-0.222	0.472	-0.470	0.638	0.837
SVSP-5	10829.466	1.964	0.726	2.707	0.007**	0.074
SVMPII-1	77902.986	0.911	0.480	1.898	0.058	0.273
BPP-1	120779.234	-0.334	2.184	-0.153	0.878	0.954
HYAL-1	3203.542	0.434	0.624	0.696	0.487	0.767
SVMPIII-1	236.935	4.758	2.234	2.130	0.033*	0.197
VEGF-1	2696.561	4.593	1.031	4.457	<0.001**	<0.001**
Waprin-1	51.896	-0.411	0.679	-0.606	0.545	NA

**Fig. 5:**
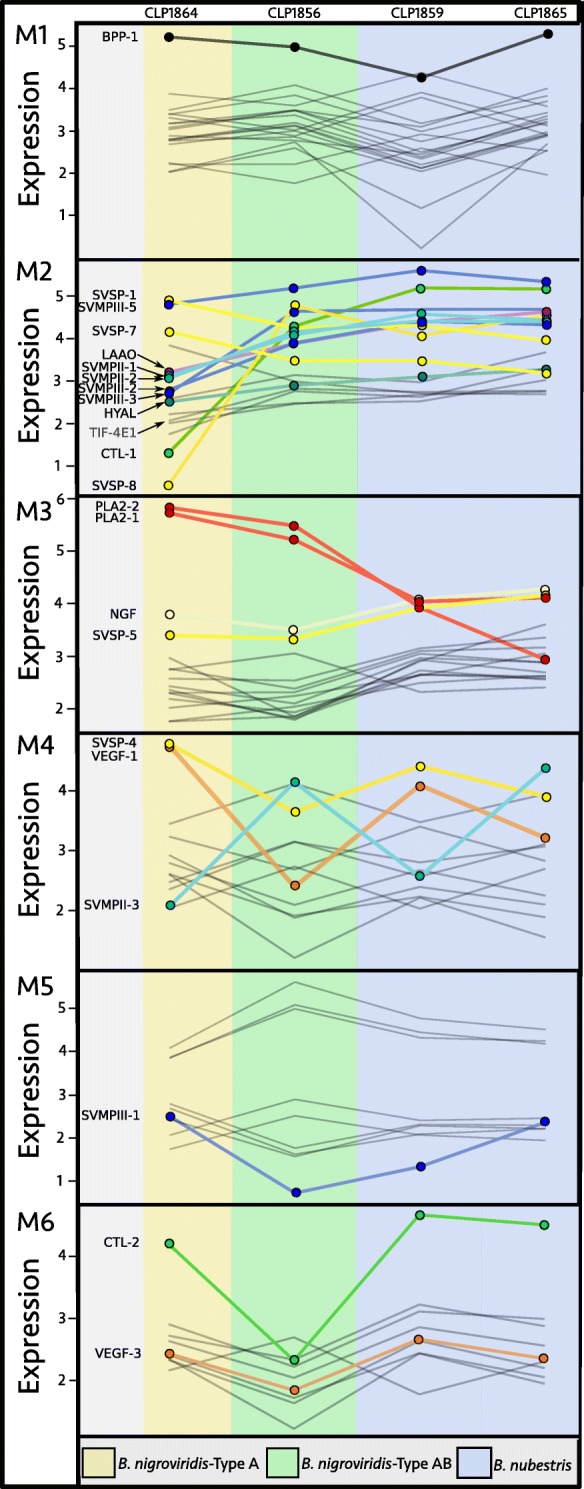
Expression profiles for the six expression modules identified by CEMiTool. Each line represents a transcript and its change in expression across treatments. Toxins assigned to each module are colored by class and labelled. Nontoxins associated with a module are shown as grey lines. Toxins generally associated with the Type A and Type B venom phenotypes (neurotoxic PLA_2_ subunits and SVMPs, respectively) largely separated into two modules: M2 and M3. *B. nigroviridis* with Type A+B venom showed generally intermediate expression of A-B associated toxins

We implemented WGCNA assigning three venom phenotypes as "treatments": Type A (*B. nigroviridis* CLP1864), Type A+B (*B. nigroviridis* CLP1856), and Type B (*B. nubestris* CLP1859 and CLP1865). After transcript filtering, 83 transcripts, including 22 toxin transcripts, were segregated into six modules (Fig. 5, in Additional file 1: Table S1). Most of the toxins associated with the Type A/Type B phenotypes segregated into two distinct modules. Module 2 contained five of the seven orthologous SVMPs while module 3 contained both nigroviriditoxin subunits. SVSPs were distributed across three modules, including module 2 and module 3. Similarly, BPPs were the only toxin assigned to module 1 which appeared to primarily capture intraspecific variation in *B. nubestris*. Of the three orthologous CTLs, one was removed during filtering and the remaining two were assigned to modules 2 and 6. Finally, two VEGFs were assigned to two separate modules as well. We did not identify any transcription factors associated with the putatively Type A or Type B modules. However, we did identify a translation initation factor, TIF-4E1, associated with module 2.

### Gene family analyses

To better understand the dynamics of transcript turnover (i.e., gene duplications and transcript losses through either gene loss or gene silencing) in relation to families associated with specific modules, we inferred toxin family phylogenies for four highly expressed and diverse toxin families and identified species-specific gene duplication and transcript loss events. As expected, our results suggest that the majority of toxin genes in *B. nigroviridis* and *B. nubestris* were likely present in their common ancestor. In three of the four toxin families, OrthoFinder identified one-to-one orthologs for the majority of toxins, although expression levels were not necessarily conserved (Fig. 5). However, each toxin family exhibited at least one species-specific toxin loss and three of the families showed evidence of both losses and duplications.

Transcript turnover was lower in families with a higher proportion of toxins sorted into a specific submodule. The two CTLs were split between two expression submodules (M2 and M6) and had four deletions and one duplication. Similarly, five SVSPs were split between three modules with three SVSPs assigned to module 2. SVMPs were inferred to have a single duplication and loss and were similarly assigned to three modules (M2, M4, and M6), though the five consistently highly expressed SVMPs were assigned to M2. PLA_2_s were the only family to experience a single species-specific toxin transcript loss, and the two orthologous toxins were assigned to M3.

**Fig. 6:**
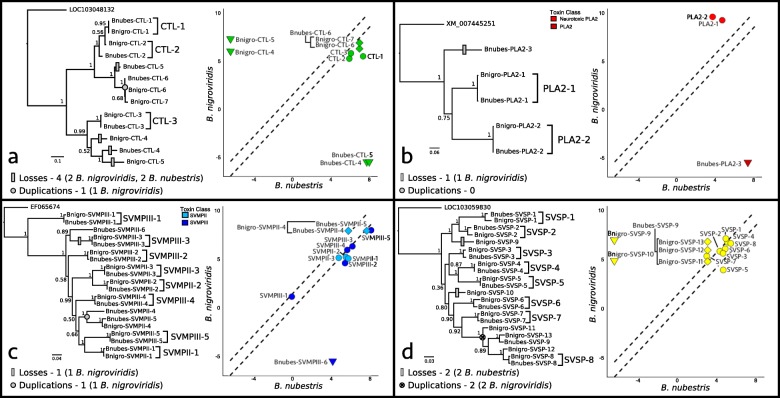
Toxin family phylogenies and expression plots of **a** C-type lectins (CTLs), **b** phospholipase A_2_s (PLA_2_s), **c** snake venom metalloproteinases (SVMPs), and (d) snake venom serine proteases (SVSPs). Single copy toxin orthologs identified by OrthoFinder are marked by brackets in the phylogeny. Toxin transcript gains and losses were inferred based on a simple parsimony model and are shown on phylogenies as grey circles and rectangles, respectively. Expression plots are based on average expression of each toxin for each species and dashed lines denote 99% confidence interval established by nontoxin expression. Identified orthologs are shown as colored circles and losses as colored inverted triangles. Duplicated toxins are shown as colored diamonds and expression of each copy is plotted against expression of their orthologous counter part in the other species (identified with bracketing on plots)

**Fig. 7:**
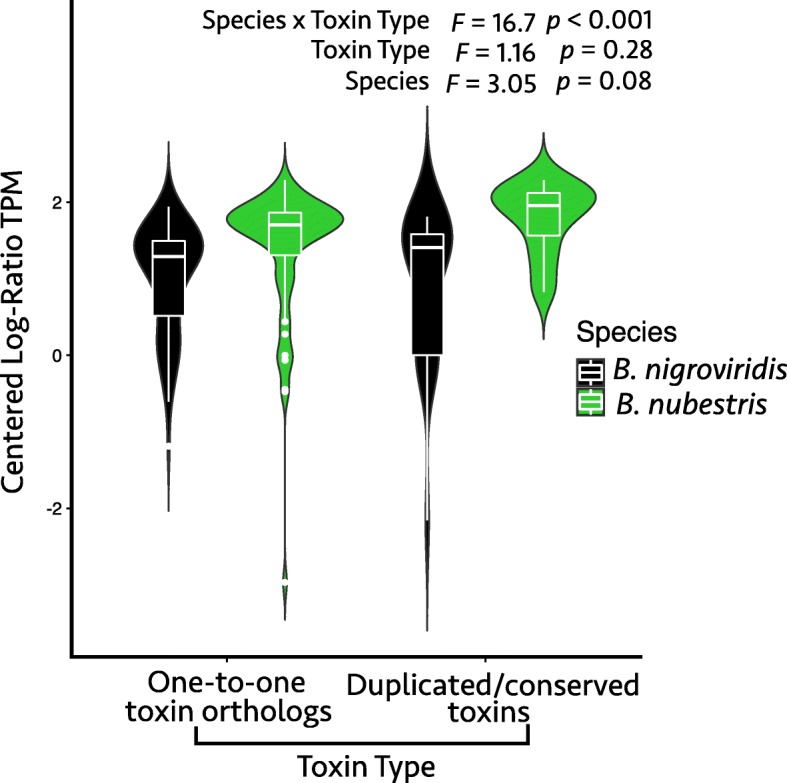
Violin plots comparing expression of orthologous and paralogous toxins for *Bothriechis nigroviridis* and *B. nubestris*. Orthologous and paralogous toxins were not differentially expressed between the species

In both SVMPs and SVSPs, we observed sequence divergence that occurred in one or more toxin copies following a duplication event (Fig. 6). In the case of SVSPs, nucleotide sequence divergence was sufficient to give conflicting phylogenetic signal when compared to the amino acid-based phylogeny inferred by OrthoFinder (Fig. 6, in Additional File 1: Figure S1). Although we did not find a significant difference in expression of one-to-one toxin orthologs compared to duplicated or conserved toxins (*p* = 0.28), we did find a marginally significant interaction between species and expression of one-to-one orthologs versus duplicated or conserved toxins (*p* = 0.08, Fig. 7). More specifically, *B. nubestris* appeared to exhibit proportionately higher expression of toxins, but also disproportionately higher expression of duplicated and conserved toxins (Fig. 7).

### Sequence based selection analyses

To determine the extent and role of sequence diversification in differentiating venoms, we compared pairwise values of *ω*, *dS*, and *dN* between toxin and nontoxin orthologs. Toxin sequences exhibited significantly higher values of *ω* (*p* <0.001) with three toxins, CTL-2, SVMPII-1, and SVMPIII-5, having *ω* values >1 indicating positive selection (Fig. 7). Despite having a higher *ω* ratio than the background nontoxins, the overall mean *ω* for toxin sequences was 0.56. Additionally, we tested for differences in synonymous and nonsynonymous substitution rates between toxins and nontoxins under the expectation that toxins and nontoxins should display similar background synonymous substitution rates but differ in nonsynonomous substitutions resulting in diversifying selection. As expected, we found no differences in synonymous substitution rates between toxins and nontoxins (*p* = 0.252) but significantly higher nonsynonymous substitution rates (*p* <0.001). Moreover, nine toxins had nonsynonymous substitution above the 95th percentile of nontoxin sequences; nearly double the number of toxins above the 95th percentile of *ω*. However, four of these toxins were found to have synonymous substitution above the 95th percentile of nontoxin sequences.

## Discussion

We tested the hypothesis that dimensions of the neurotoxic-hemorrhagic venom phenotype were associated with specific submodules of toxin expression. We identified six submodules of expression variation, which included a primarily Type A submodule containing both nigroviriditoxin homolog subunits and a primarily Type B submodule containing the majority of orthologous SVMPs. The findings supported our hypothesis and implicate submodular regulation as a mechanism for rapid venom diversification. Modular expression regimes would allow rapid transitions between phenotypes while avoiding or minimizing occurrence of low-fitness intermediates [2] and facilitate ontogentic shifts observed in many groups [27, 28, 43, 44]. In the *Bothriechis* system, modularity effectively explains many of the toxin expression differences between *B. nigroviridis* and *B. nubestris*. The patterns of modularity observed here are also consistent with on-going genomic research to elucidate the genomic architecture mediating venom phenotype evolution [15, 45, 46]. Taken together these findings provide strong support for a role of sub-modular variation mediating changes in snake venom phenotypes.

### Modularity underlying the neurotoxic-hemorrhagic dichotomy

The patterns of modularity and submodular organization inferred by WGCNA analyses explained much of the inter- and intraspecific variation in toxin expression we observed for *B. nigroviridis* and *B. nubestris*. We recovered a venom gland transcriptome for the northern *B. nigroviridis* consistent with the published proteomic venom phenotype and Type A venom expression. The increase in expression of nigroviriditoxin/nigroviriditoxin homologs is accomplished primarily through modification of regulatory patterns in module 3. Similarly, modifications to regulatory elements in module 2 can mediate expression regime shifts of many toxins, especially SVMPs. The strong association of these modules with species-specific patterns of inheritance demonstrate how modularity can promote rapid phenotypic transition among recently diverged and/or eco-morphically conserved species.

Of note was the Type A+B expression pattern in the southern *B. nigroviridis* which suggested intermediate or combined expression of the Type A and Type B submodules. Although Type A+B venoms have been documented in multiple species [19, 39] they are primarily associated with species exhibiting population level neurotoxic-hemorrhagic dichotomies and often occur at lower frequency than either the Type A or Type B phenotypes [11]. If this pattern holds true in *B. nigroviridis*, it would suggest the existence of individuals or populations of *B. nigroviridis* that have primarily Type B venom. Population level sampling has been difficult to attain due to the inherent rarity of this species and the logistical challenges of sampling many of the undisturbed, high-elevation regions of its distribution. However, population level sampling will be key for understanding the ecological and evolutionary dynamics of venom variation in this species. More importantly, the occurrence of the Type A+B phenotype in *B. nigroviridis* and other species suggests that the Type A and B submodules are not mutually exclusive. Rather, each module likely has independent genetic architectures which can occur in variable combinations among populations and species.

Modular expression effectively explains Type A/Type B toxin variation among these two species, but several toxin families such as CTLs, SVSPs, and VEGFs did not fit this framework. The variation observed in these families underscores the diversity of expression patterns in venom toxins and presents an ongoing challenge for the future. Although a great deal of work has been devoted to dissecting broad patterns of venom variation (e.g., neurotoxic-hemorrhagic dichotomy), the mechanisms influencing variation in other diverse toxin families such as SVSPs and CTLs deserves further investigation.

While our findings present evidence for submodularity of toxin expression, it is important to note their limitations as well. WGCNA identifies submodular clusters based on positive and negative correlations in transcript expression across assigned treatments with the expectation that these transcripts may be influenced by common regulatory elements. Because coexpression network analyses are based on observed patterns of expression rather than experimental validation, they are better regarded as hypotheses of submodular association rather than empirical findings. Moreover, WGCNA are ideally implemented using thousands of candidate transcripts derived from thoroughly assembled and annotated genomes with tens of replicates across treatments for robust inference. Unfortunately, genomic resources remain limited for snakes and such large sample sizes are difficult to attain for many species. Here, we have implemented WGCNA with a much reduced sample size and far fewer candidate genes than is typically ideal, which may make module assignment less powerful and robust, especially for lowly expressed transcripts. Nevertheless, our analyses assigned many highly expressed toxins to biologically plausible submodules corresponding to known axes of phenotypic variation in snake venom. Thus, we believe that WGCNA as implemented here represent an important proof-of-concept in the relevance and potential of these methods and the conceptual framework of modularity for evolutionary study of venom differentiation.

### Mechanisms promoting modularity

Although our WGCNA and similar approaches identify submodules of variation based on phenomenological rather than mechanistic models, observed patterns of expression and recent genomic work implicate several general mechanisms contributing to modularity of the system. For instance, one of the primary advantages of co-expression network approaches is the ability to identify regulatory components such as transcription factors that potentially mediate the identified expression differences. In sub-module 2, we identified one translation initiation factor that showed increased expression with progression towards the Type B phenotype. Translation initiation factors enhance translation by stabilizing mRNA and facilitating assembly of ribosomal complexes [47]. In mammals, TIF-4E is required for efficient translation and acts as a translational regulatory mechanism [47]. Here, its association with module 2 may reflect an effort to promote rapid translation of the relatively large and highly expressed SVMPs. Though concordant expression of TIF-4E and module 2 toxins does not necessarily imply a causative link, it does present a hypothesis to test through functional validation.

The identification of primarily neurotoxic and hemorrhagic submodules are also consistent with recent genomic evidence which show that Type A and Type B toxins are inherited as independent haplotypes [15, 45, 46]. In some cases, presence and absence differences in these genes have been implicated as the primary drivers of variance in Type A/Type B phenotypes. In the case of the northern *B. nigroviridis*, absence of the SVMP tandem array could account for both the low expression of SVMPs and their inferred absence from the transcriptome (Table 2). In contrast, both *B. nubestris* individuals express low levels of a nigroviriditoxin homolog. Despite patterns of low expression, the sequences of the *B. nubestris* PLA_2_s were highly conserved with respect to nigroviriditoxin; both subunits had over 99% nucleotide sequence similarity with three nonsynonomous substitutions occurring in the beta subunit and one synonymous substitution occurring in the alpha subunit. The conservation of these sequences suggests that the *B. nubestris* variants of nigroviriditoxin have likely retained their neurotoxic function and that convergence on a "low neurotoxicity" phenotype therefore occurs through regulatory evolution in *Bothriechis* rather than through gene loss/gain as is observed in other species [15, 45, 46].

If expression patterns of the Type A and Type B submodules are inherited as independent haplotypes with additive effects, we can hypothesize that combined phenotypes are possible and should exhibit intermediate expression of of each module. The expression patterns apparent in the southern *B. nigroviridis* support these predictions as it displayed intermediate expression between the Type A *B. nigroviridis* and the Type B *B. nubestris* for the majority of Type A and Type B associated toxins. Additive expression of species-specific toxins has also been observed in interspecific hybrids where the putatively heterozygous offspring exhibit lower expression levels than presumably homozygous parents [35]. In the case of *B. nigroviridis*, intermediate expression observed in the southern *B. nigroviridis* could feasibly be the result of heterozygosity at Type A and Type B loci, though such a hypothesis is largely postulation without genomic evidence. As such, comparative genomics approaches that test architectural mechanisms promoting and mediating modularity are a promising avenue for future work.

### Transcript turnover and diversification in a modular system

We expected selective optimization for modularity of toxin expression to affect toxin transcript turnover and sequence diversification. We tested for these effects in four toxin families and found that although all four toxin families had experienced some turnover, rates of duplication and loss were higher in toxins less associated with specific modules. Many snake toxin families have experienced dramatic expansions since their common ancestor [9] though the frequency of toxin duplications and losses within species is not clear. The marginal decrease in transcript-turnover with increased association with a specific submodule suggests selection for maintaining these toxins. Duplications are often implicated as having a primary role in toxin neofunctionalization by creating functional redundancy that allows toxins to ‘explore’ the phenotype space [9, 48, 49], but can also occur as a mechanism to increase expression of beneficial toxins [50]. We observed both increased sequence divergence following duplication and a marginal increase in expression of duplicated or conserved (i.e., not deleted or silenced) toxins specific to the *B. nubestris* lineage. Whether the possible emphasis on expression of paralogous versus orthologous toxins reflect phenomena unique to the *B. nubestris* lineage or a broader trend in the evolution of the more complex, hemorrhagic venom types is not clear, especially given our limited sample size. However, increased sampling of lineages and their toxin compositions will provide improved resolution to test the extent and role of gene duplication and loss in venom diversification.

We expected sequence diversification to be lowest in module associated toxins, but we did not find evidence to support this. Two of the three toxins with *ω* above one were SVMPs associated with Module 2, suggesting that although regulation may be conserved/coordinated, functionality is not. Many of the toxins with elevated rates of nonsynonymous substitution had similarly high rates of synonymous substitutions, which may indicate an overall higher substitution rate than the genomic background. Notably, SVSPs, which were generally less associated with a specific module, displayed some of the highest values of both *dN* and *dS*. The overall elevated substitution rates of these toxins and the lack of correspondence to clear expression regimes may reflect higher rates of substitution and recombination in these gene regions, though patterns of gene expression and the organization of the genetic architecture of SVSP regions is not well understood. Overall, toxin *ω* values were generally below what is expected under positive selection with just a few toxins displaying *ω* values greater than 1. Instead, toxin evolution between species appears to function under a model of relaxed purifying selection, which has been similarly noted in other interspecific comparisons of toxin sequence evolution [20].

## Conclusions

Snake venoms are key innovations that have allowed the diversification of species across the globe. Unfortunately, many of the genomic mechanisms governing rapid variation of phenotypes remain uncertain. Through comparative transcriptomics and coexpression network analyses, we demonstrated how rapid transition between a common phenotypic venom dichotomy can occur through submodular regulation of the associated toxins. Modularity of the venom system and submodular variation of venom classes likely contribute to broader patterns of variation observed across taxonomic levels [51]. As genomic and transcriptomic resources become more available for venomous snakes, systems-based approaches such as the co-expression network analyses used here will yield more comprehensive understanding of the evolution of venoms and other complex, modular traits. Although our work presents these findings in the limited context of a single species pair, it highlights the importance of considering how complex traits function and evolve as a modular system. Our understanding of the selective forces that generate modularity and how modularity in turn mediates and facilitates the evolution of complex traits remains incomplete. However, as we have shown here, on-going efforts to address these questions in dynamic adaptive systems can provide key insights that lead to a more integrated understanding of the genomics of rapid adaptation in complex traits.

## Methods

### Sample collection

**Fig. 8:**
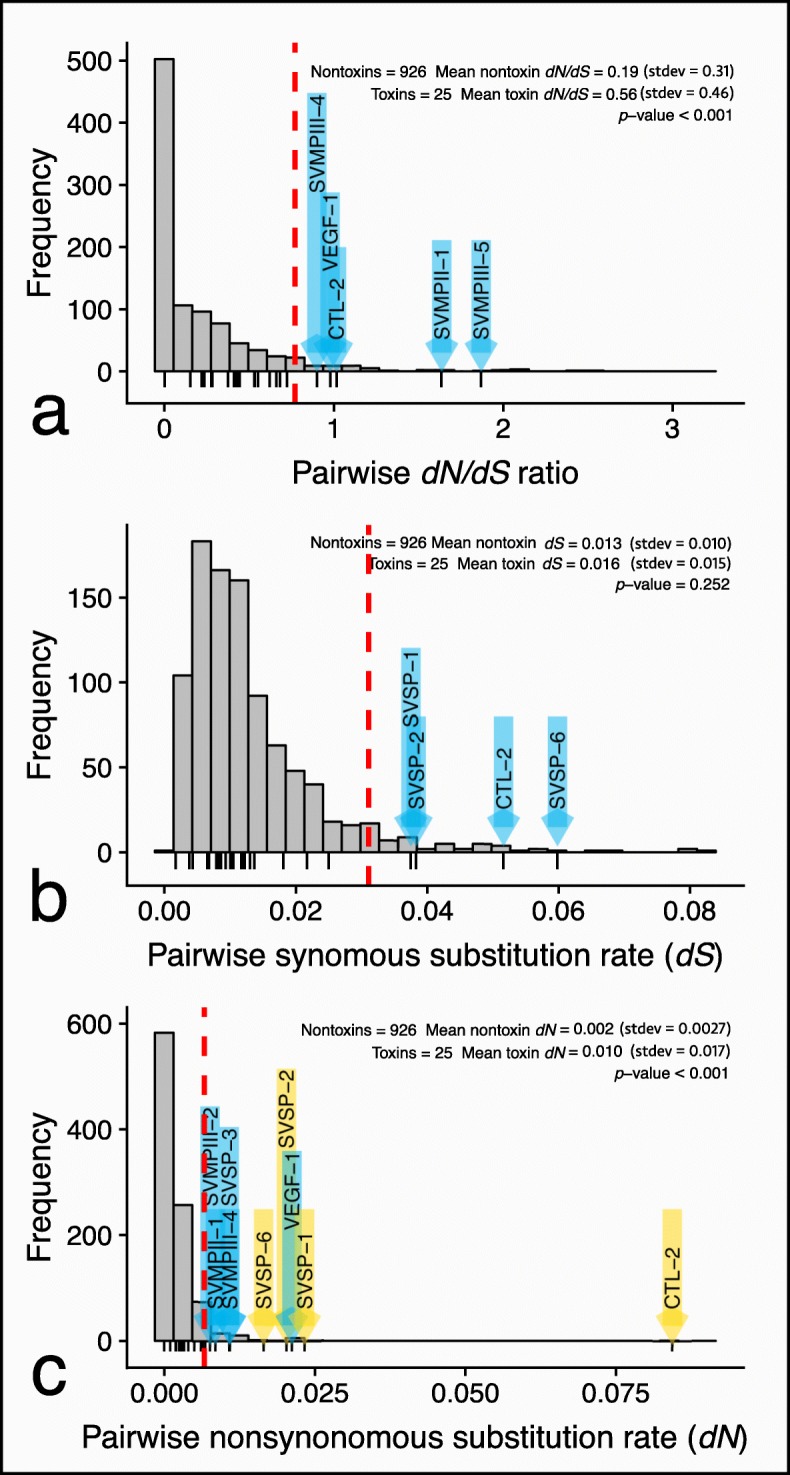
Distribution of **a** pairwise *dN/dS* ratios, **b** synonymous substitution rates, and **c** nonsynonymous substitution rates of orthologous transcripts. Dashed red lines denote 95 percentiles based on distribution of nontoxins. Lines beneath plots indicate toxins, and toxins with values greater than the 95 percentile are marked with blue arrows. In **c**, toxins above the 95th percentile with elevated synonymous mutation rates (i.e., above the 95th percentile in **b** are colored yellow. Toxins had statistically higher *dN/dS* ratios and nonsynonymous substitution rates based on a Wilcoxon signed rank test. Toxin and nontoxin synonymous mutation rates were not significantly different

We collected two individuals of *Bothriechis nigroviridis* and two *B. nubestris* in May-June of 2016 for venom gland extraction and sequencing. Due to the smaller range of *B. nubestris*, both individuals were collected from the same locality (∼1 km apart), San Gerardo de Dota, San Jose province, Costa Rica. *Bothriechis nigroviridis* occupies a wider range than *B. nubestris* and we collected two individuals from distant populations. One of these individuals (CLP1864), was collected from outside of the La Esperanza sector of Parque Tapanati, Cartago province, Costa Rica, a locality that is ∼50 km south of specimens collected and used in previous proteomic studies characterizing the venom of this species [30]. The second individual (CLP1856) came from the southern most portion of the species’ range in Costa Rica, Las Tablas, Puntarenas province, Costa Rica (Fig. 8) ∼200 km southeast of specimens used in [30].

Following collection, each individual had its venom collected via manual extraction. Collected venoms were lyophilized and stored at -20 C for later use. Each animal was sacrificed four days later when transcription of venom proteins is at its maximum [52], via injection of sodium pentobarbitol (100mg/kg). Venom glands were dissected and stored separately in approximately 2 mL of RNAlater preservative. Animal carcasses were preserved as museum specimens with 10% buffered formalin and deposited in the Universidad de Costa Rica. The above methods were approved by University of Central Florida Institutional Animal Care and Use Committee (IACUC) protocol 16-17W, Clemson University IACUC protocol number 2017-067, and Universidad de Costa Rica Comimté Institucional para el Cuidado y Uso de los Animales (CICUA) permit number CICUA-082-17.

### Venom gland transcriptome sequencing

Total RNA was extracted from left and right glands independently using a standard, Trizol reagent extraction as described in [53]. Briefly, diced venom gland tissues were submerged in 500 *μ*L of Trizol, homogenized with a sterile 20-gauge needle, and treated with an additional 500 *μ*L of Trizol and 200 *μ*L chloroform. RNA was then separated from tissue, cellular components, and DNA by centrifuging the total mix in a 5Prime phase lock gel heavy tube for 20 minutes at 12,000 g. Supernatant containing the RNA was transferred to a new tube and RNA was precipitated with 500 *μ*L of isopropyl alcohol. Pelleted RNA was washed in 75% ethanol and re-suspended in RNAase free water. Extracted total RNA was checked for quality and quantified using either an Agilent 2100 Bioanalyzer or Agilent 2200 TapeStation and stored at -80 C.

We prepared cDNA libraries from 1 *μ*L high quality total RNA using the NEBNext Ultra RNA library Prep Kit for Illumina following the manufacturer’s instructions. Specifically, we isolated polyadenalated RNA with the NEB Poly(A) Magnetic Isolation Module (New England Biolabs) and fragmented resulting mRNA by heat fragmentation at 70 ^∘^ C for 14.5 minutes to attain an average size of approximately 370 bp. mRNA fragments were reverse transcribed to cDNA and each library was ligated with a unique combination of index primers and Illumina adapters. The cDNA libraries were amplified through PCR using the NEBNext High-Fidelity 2X Hot Start PCR Master Mix and 14 cycles of PCR. Amplified cDNA was purified with Agencourt AMPure XP PCR Purification beads. The resulting libraries were checked for quality, fragment size distribution, and concentration on either an Agilent 2100 Bioanalyzer or Agilent 2200 TapeStation. KAPA qPCR was additionally performed on each sample library to determine amplifiable concentrations. Libraries were then pooled in groups of twelve with equal representation of amplifiable cDNA for sequencing.

Sequencing took place on an Illumina HiSeq 2000 at the Florida State University College of Medicine’s Translational Science Laboratory. Combined libraries were multiplexed and sequenced with a 150 bp paired-end rapid run lane. Raw reads were demultiplexed and quality checked in FastQC [54]. To account for reads which may have been mis-assigned during demultiplexing, we used jellyfish v.2.2.6 [55] and KAT v.2.3.4 [56] to identify and filter reads with kmers that exhibited more than a 500 fold difference in occurrence between samples sequenced on the same lane. Adapter sequences and low quality bases were then trimmed using trim-galore v.0.4.4 [57]. Finally, to increase both quality and total length of read sequences, we used PEAR v 0.9.6 [58] to merge paired reads with a 3’ overlap of greater than 10 bp.

### Transcriptome assembly and analyses

Previous transcriptome studies have shown the challenges associated with venom gland transcriptome assembly, due to the contrast in a proportionately low number of highly expressed toxin transcripts compared to the much broader, low expression of house keeping genes [59]. To overcome this, we performed three independent assemblies using Extender [53], the DNAstar NGen assembler v.15.0, and Trinity v.2.4.0 [60] per the strategy suggested in Holding et al. [59]. Sequence identities of toxins from each assembly were identified via local blastx search of SWISS-prot’s curated toxins database. Contigs with a blast match of greater than 90% identity were then clustered against a database of identified snake toxins to annotate coding regions of 90% similarity or greater. Coding regions of remaining toxin contigs were annotated manually in Geneious v.10.2.3 [61]. Contigs which were not identified as toxins were annotated by clustering against a database of previously identified snake nontoxins to annotate coding regions of 90% similarity or greater representing nontoxin transcripts used in later analyses. Annotated transcripts from independent assemblies were combined and duplicate sequences as well as coding regions with ambiguous sites were removed. The remaining transcripts were screened for chimeric or mis-assembled coding sequences by mapping merged reads against these sequences with bwa v.0.7.16 [62] and checking for uneven read distribution across sites. Specifically, sequences with sites where the mean number of bases per read on either side of a site differed by more than 50% of the mean read length were considered likely chimeras, checked manually, and removed accordingly. We clustered the remaining transcripts at a threshold of 98% similarity to account for toxin alleles or recent paralogs that may be present. This represented the final transcriptome for each individual. To account for variation among individuals in a species and for stochastic variation in the assembly process that may have resulted in failure to assemble specific toxins in a given individual, we combined final contig sets for individuals of the same species, removed duplicates, and clustered coding regions of 98% similarity to create a master transcriptome for each species. These species-specific master transcriptomes were then used for subsequent read mapping and expression analyses.

### Expression analyses and ortholog identification

To determine relative expression of transcripts, we mapped reads from individuals to their species master transcriptome with Bowtie2 v2.3.2 and calculated relative expression with RSEM v.1.3.0 [63]. Intraspecific differences in expression were assessed using species-specific datasets for *B. nigroviridis* and *B. nubestris*. Because our limited intraspecific sampling precluded formal tests for differential expression within species, we generated pairwise null distributions of expression divergence for each species based on nontoxin expression to identify outlier toxins similar to [64]. Data were first centered log-ratio (clr) transformed to normalize the expression distributions while accounting for the compositional nature of relative expression values (e.g., TPM) using the cmultRepl function in the R package zCompositions [25, 65, 66]. Toxins whose pairwise divergence in expression fell outside the 99th percentile of the centered log-ratio transformed nontoxin distributions were considered outliers that are likely differential expression. RSEM can assign non-zero values to transcripts that may not be present in the transcriptome through mis-mapping of reads from other transcripts with regions of high similarity. To verify the extent to which toxins varied in presence or absence within species we aligned merged reads to the species-specific transcript sets to screen for poor read mapping. Toxins that had regions greater than 10% of the total sequence length with less than 5x coverage or highly anomalous read distributions (determined by manual review) were considered absent in the transcriptome of a given individual.

Toxin families in snakes are notorious for undergoing rapid expansions and losses, which is problematic for interspecific comparisons which assume orthology among matched transcripts. To overcome this we identified orthologous groups of transcripts using OrthoFinder v.2.3.1 [42] specifying multisequence alignments with mafft. OrthoFinder identifies groups of sequences derived from a single gene in the common ancestor of compared species (i.e., orthogroups), as well as identifies conserved orthologs within orthogroups. We classified transcripts as orthologs or paralogs by parsing the OrthoFinder “orthologs" output to identify single copy orthologs and one-to-one orthologs within orthogroups using a custom python script (orthocombiner.py). For interspecific comparisons, expression data for orthologous and paralogous transcripts were combined into a single dataset where paralogous transcripts were given an expression value of zero where absent for a given species. We used estimates of read counts from RSEM to test for differences in transcript expression with DESeq2 in R v.3.5.3 [67].

### Network analyses

We performed weighted gene coexpression network analysis using the R package CEMitool [68] in R. A variance stabilizing transformation (vst) was used and transcripts were filtered to reduce correlation between variance and gene expression. We used pearson’s coefficient as the correlation method and a beta value of 10 was automatically selected. The minimum module size was set to 1 to allow the greatest flexibility in identifying modules of correlated expression. Because of the high variability in venom composition observed among *B. nigroviridis* (see above), we annotated samples as one of three venom types which correspond to venom phenotypes observed in rattlesnakes: *B. nigroviridis* Type A (CLP1864), *B. nigroviridis* Type A+B (CLP1856), and *B. nubestris* type B (CLP1859 and CLP1865).

### Gene family analyses

To more closely examine how toxin family expansion, duplications, and loss have shaped venom composition, we constructed phylogenies for the four most highly expressed toxin families: C-type lectins (CTLs), PLA_2_s, snake venom serine proteases (SVSPs), and SVMPs. Alignments for each family were generated with mafft v.7.407 [69] and checked manually in Geneious. Partitioning schemes for each gene family were determined using PartitionFinder v.2 [70]. Phylogenies were then recovered with MrBayes v.3.2.6 [71]. MrBayes was run using one cold and three heated chains for 10 million generations with a variable rate prior. We then identified and mapped species-specific deletion and duplication events onto the trees based on the output of OrthoFinder. We considered toxins that were unassigned an ortholog to be indicative of gene loss in one species while one to many ortholog assignments indicated duplications within a species. We tested for differences in expression of one-to-one orthologs versus conserved and duplicated toxins with a two-way factorial with toxin type and species as factors in R. TPM values were used as the metric for expression and were centered log-ratio transformed to linearize the data while preserving their compositional nature [25, 65].

### Sequence analyses

We compared divergence of orthologous toxin and nontoxin transcripts by calculating *dN/dS* ratios (*ω*). Orthologous transcripts were first aligned by codon using PRANK v.170427 [72]. PRANK alignments were then used as input to estimate *ω*, *dS*, and *dN* with codeml in paml v. 4.9 [73].

We compared *ω*, *dS*, and *dN* of toxin genes against a background of nontoxins as in [20] to discern if toxin genes exhibited higher synonymous and/or nonsynonymous substitution rates and if toxins displayed high rates of positive selection (i.e., higher values of *ω*). We excluded sequences with *dS* <0.001 due to the possibility of estimating excessively inflated values of *ω*, and sequences with *dS* >0.10 to reduce the risk of including misidentified orthologs. Statistical differences in *ω*, *dS*, and *dN* values between toxins and nontoxins were tested with a wilcoxon sign rank test in R.

## Supplementary information

**Additional file 1** This file contains supplemental table S1 and supplemental figure S1. Supplemental table S1 denotes module assignment of all coding sequence used in WGCNA analyses. Supplemental figure S1 shows phylogenetic trees of orthogroups containing toxins derived from amino acid alignments of coding sequences by OrthoFinder.

## Data Availability

Raw sequence data and transcript sequences generated during the current study are available on the National Center for Biotechnology Information (NCBI) under accession numbers given in Table 1. Consensus transcriptomes have been to submitted to the NCBI Transcriptome Shotgun Assembly (TSA) database under GIBL00000000 (*Bothriechis nigroviridis*) and GIBM00000000 (*B. nubestris*). Scripts used in data analyses are available on GitHub at: https://github.com/masonaj157/Trait_differentiation_and_modular_expression_in_palm-pitvipers

## Abbreviations

BPP: Bradykinin potentiating peptide
CTL: C-type lectin
PLA_2_: Phospholipase A_2_
SVMP: Snake venom metalloproteinases
SVSP: Snake venom serine proteinase
TIF: Translation initiation factor
VEGF: Snake venom vascular endothelial growth factor
WGCNA: Weighted gene coexpression network analysis
