# Supplementary material for: Trait differentiation and modular toxin expression in palm-pitvipers
Source: BMC Genomics. 2020 Feb 11;21:147. doi: 10.1186/s12864-020-6545-9 (PMC7014597; doi:10.1186/s12864-020-6545-9)
Supplement: Supplementary file 1 — Additional file 1 This file contains supplemental table S1 and supplemental figure S1. Supplemental table S1 denotes module assignment of all coding sequence used in WGCNA analyses. Supplemental figure S1 shows phylogenetic trees of orthogroups containing toxins derived from amino acid alignments of coding sequences by OrthoFinder. [file 12864_2020_6545_MOESM1_ESM.docx]

*BMC Genomics*

**SUPPLEMENTAL MATERIAL**

**Trait differentiation and modular toxin expression in Palm-Pitvipers**

Andrew J. Mason, Mark J. Margres, Jason L. Strickland, Darin R. Rokyta, Mahmood Sasa, and Christopher L. Parkinson

Table S1. Module assignment for orthologous transcripts from *Bothriechis nigroviridis* and *B. nubestris* passing VST transformation and filtering by CEMiTool.

| Module | *B. nubestris* genes | *B. nigroviridis* genes |
| --- | --- | --- |
| M1 | Bnubes-BPP-1 | Bnigro-BPP-1 |
| M1 | Bnubes-GRP78 | Bnigro-GRP78 |
| M1 | Bnubes-Parvalbumin | Bnigro-Parvalbumin |
| M1 | Bnubes-Galectin1 | Bnigro-Galectin1 |
| M1 | Bnubes-MyosinLight | Bnigro-MyosinLight |
| M1 | Bnubes-Endoplasmin | Bnigro-Endoplasmin |
| M1 | Bnubes-PDI-A4 | Bnigro-PDI-A4 |
| M1 | Bnubes-Peroxiredoxin6 | Bnigro-Peroxiredoxin6 |
| M1 | Bnubes-CreatineKin | Bnigro-CreatineKin |
| M1 | Bnubes-Actin-a | Bnigro-Actin-a |
| M1 | Bnubes-TroponinC | Bnigro-TroponinC |
| M1 | Bnubes-TF-LIM-LMO4 | Bnigro-TF-LIM-LMO4 |
| M1 | Bnubes-Calglandulin | Bnigro-Calglandulin |
| M1 | Bnubes-Calmod | Bnigro-Calmod |
| M1 | Bnubes-TropI | Bnigro-TropI |
| M1 | Bnubes-AdenylosuccinateLyase | Bnigro-AdenylosuccinateLyase |
| M1 | Bnubes-Rab-18 | Bnigro-Rab-18 |
| M1 | Bnubes-TIF-1b | Bnigro-TIF-1b |
| M1 | Bnubes-PepMethSulfRed | Bnigro-PepMethSulfRed |
| M1 | Bnubes-SC35B1 | Bnigro-SC35B1 |
| M2 | Bnubes-SVMPIII-5 | Bnigro-SVMPIII-5 |
| M2 | Bnubes-CTL-1 | Bnigro-CTL-1 |
| M2 | Bnubes-SVMPIII-3 | Bnigro-SVMPIII-3 |
| M2 | Bnubes-SVSP-1 | Bnigro-SVSP-1 |
| M2 | Bnubes-SVSP-8 | Bnigro-SVSP-8 |
| M2 | Bnubes-SVMPII-1 | Bnigro-SVMPII-1 |
| M2 | Bnubes-LAAO-1 | Bnigro-LAAO-1 |
| M2 | Bnubes-SVMPII-2 | Bnigro-SVMPII-2 |
| M2 | Bnubes-SVMPIII-2 | Bnigro-SVMPIII-2 |
| M2 | Bnubes-SVSP-7 | Bnigro-SVSP-7 |
| M2 | Bnubes-ErythMem | Bnigro-ErythMem |
| M2 | Bnubes-d3PGDH | Bnigro-d3PGDH |
| M2 | Bnubes-HYAL-1 | Bnigro-HYAL-1 |
| M2 | Bnubes-SPARC | Bnigro-SPARC |
| M2 | Bnubes-TIF-4E1 | Bnigro-TIF-4E1 |
| M2 | Bnubes-ATOX1 | Bnigro-ATOX1 |
| M2 | Bnubes-EpididymalSecretory-E1 | Bnigro-EpididymalSecretory-E1 |
| M3 | Bnubes-PLA2-2 | Bnigro-PLA2-2 |
| M3 | Bnubes-PLA2-1 | Bnigro-PLA2-1 |
| M3 | Bnubes-NGF-1 | Bnigro-NGF-1 |
| M3 | Bnubes-SVSP-5 | Bnigro-SVSP-5 |
| M3 | Bnubes-GST-t1 | Bnigro-GST-t1 |
| M3 | Bnubes-poly-rC-2 | Bnigro-Vigilin-1 |
| M3 | Bnubes-Translocator | Bnigro-Translocator |
| M3 | Bnubes-SuperoxideDismutase | Bnigro-SuperoxideDismutase |
| M3 | Bnubes-NucGTP1 | Bnigro-NucGTP1 |
| M3 | Bnubes-GlutamineSyn | Bnigro-GlutamineSyn |
| M3 | Bnubes-MatrixGla | Bnigro-MatrixGla |
| M3 | Bnubes-Tetraspanin31 | Bnigro-Tetraspanin31 |
| M3 | Bnubes-Leydig | Bnigro-Leydig |
| M3 | Bnubes-RAB11a-2 | Bnigro-RAB11a-2 |
| M3 | Bnubes-AffCuUp2 | Bnigro-AffCuUp2 |
| M3 | Bnubes-selenoproteinU | Bnigro-selenoproteinU |
| M4 | Bnubes-SVSP-4 | Bnigro-SVSP-4 |
| M4 | Bnubes-VEGF-1 | Bnigro-VEGF-1 |
| M4 | Bnubes-SVMPII-3 | Bnigro-SVMPII-3 |
| M4 | Bnubes-Actin2 | Bnigro-Actin2 |
| M4 | Bnubes-CREGF | Bnigro-CREGF |
| M4 | Bnubes-OrnithinAminoTrans | Bnigro-OrnithinAminoTrans |
| M4 | Bnubes-tom5 | Bnigro-tom5_trinContig36585 |
| M4 | Bnubes-Tetraspanin13_trinContig2931 | Bnigro-Tetraspanin13 |
| M4 | Bnubes-IPDPDIsomerase | Bnigro-IPDPDIsomerase |
| M4 | Bnubes-Bifunctional-PURH | Bnigro-Bifunctional-PURH |
| M4 | Bnubes-C19orf60 | Bnigro-C19orf60 |
| M4 | Bnubes-DehydrocholesterolRed | Bnigro-DehydrocholesterolRed |
| M5 | Bnubes-Plasminogen | Bnigro-Plasminogen |
| M5 | Bnubes-LOC100329601 | Bnigro-LOC100329601 |
| M5 | Bnubes-HypPro1 | Bnigro-HypPro1 |
| M5 | Bnubes-AminopeptidaseA | Bnigro-AminopeptidaseA |
| M5 | Bnubes-FAM3D | Bnigro-FAM3D |
| M5 | Bnubes-Reticulocalbin1 | Bnigro-Reticulocalbin1 |
| M5 | Bnubes-Thiolase | Bnigro-Thiolase |
| M5 | Bnubes-GILT | Bnigro-GILT |
| M5 | Bnubes-SVMPIII-1 | Bnigro-SVMPIII-1 |
| M6 | Bnubes-CTL-2 | Bnigro-CTL-2 |
| M6 | Bnubes-MannosidaseIB | Bnigro-MannosidaseIB |
| M6 | Bnubes-MCFD2 | Bnigro-MCFD2 |
| M6 | Bnubes-CytC | Bnigro-CytC |
| M6 | Bnubes-VEGF-3 | Bnigro-VEGF-3 |
| M6 | Bnubes-FKBP7 | Bnigro-FKBP7 |
| M6 | Bnubes-Neurocalcin-d | Bnigro-Neurocalcin-d |
| M6 | Bnubes-mRpL55 | Bnigro-mRpL55 |
| M6 | Bnubes-InsulinInd1 | Bnigro-InsulinInd1 |

Figure S1. Toxin orthogroup trees inferred by OrthoFinder from amino acid sequences of *Bothriechis nigroviridis* and *B. nubestris* transcripts
